# Mathematical Analysis of Non-Newtonian Blood Flow in Stenosis Narrow Arteries

**DOI:** 10.1155/2014/479152

**Published:** 2014-12-17

**Authors:** Somchai Sriyab

**Affiliations:** Department of Mathematics, Chiang Mai University, Chiang Mai 50200, Thailand

## Abstract

The flow of blood in narrow arteries with bell-shaped mild stenosis is investigated that treats blood as non-Newtonian fluid by using the K-L model. When skin friction and resistance of blood flow are normalized with respect to non-Newtonian blood in normal artery, the results present the effect of stenosis length. When skin friction and resistance of blood flow are normalized with respect to Newtonian blood in stenosis artery, the results present the effect of non-Newtonian blood. The effect of stenosis length and effect of non-Newtonian fluid on skin friction are consistent with the Casson model in which the skin friction increases with the increase of ither stenosis length or the yield stress but the skin friction decreases with the increase of plasma viscosity coefficient. The effect of stenosis length and effect of non-Newtonian fluid on resistance of blood flow are contradictory. The resistance of blood flow (when normalized by non-Newtonian blood in normal artery) increases when either the plasma viscosity coefficient or the yield stress increases, but it decreases with the increase of stenosis length. The resistance of blood flow (when normalized by Newtonian blood in stenosis artery) decreases when either the plasma viscosity coefficient or the yield stress increases, but it decreases with the increase of stenosis length.

## 1. Introduction

Stenosis arteries are a narrowing or constriction of inner surface (lumen) of arteries. It is a main cause of well-known serious diseases such as atherosclerosis and cardiovascular disease to name a few (see [1, 2]). Therefore, the study of blood flow in a stenosis artery is useful for the understanding of circulatory disorders. Blood behaves as Newtonian fluid when blood flows through larger diameter arteries at high shear rate, but it exhibits a non-Newtonian fluid when it flows through small diameters arteries at low shear rate [3–5].

The Casson fluid model is a non-Newtonian fluid and widely used for blood flow in narrow arteries, for example, in [6–10]. Kuang and Luo have proposed the K-L model as an improvement of Casson model. This model is more effective in the describing of a non-Newtonian blood flow because it contains two parameters such as the yield stress and the plasma viscosity but Casson model is considered only yield stress. K-L model is more effective in describing the shear thinning behavior of blood within a wide shear rate [11]. Asharafizaadeh and Bakhshaei used the K-L model with the Lattice Boltzmann simulation [12]. Zhang and Kuang indicated that the K-L model is in good agreement with hemorheological characteristics of human [13]. Another model with two parameters, the yield stress and power law index, namely, H-B model, has also been proposed by Herschel-Bulkley; see [14, 15].

In our work, a mathematical model is developed to analyze the blood flow at low shear rate in narrow arteries with mild bell-shaped stenosis. We treated the blood as non-Newtonian by using the K-L model and discussed the effect of various parameters on the physiologically important flow quantities such as flow rate, skin friction, and resistance of blood flow.

## 2. Mathematical Formulation

We consider an axially symmetric, laminar flow and non-Newtonian incompressible viscous blood in the *z*-axial direction through a circular artery. The bell-shaped mild stenosis in arrow artery is studied and the artery wall is assumed to be rigid. Many researchers studied the non-Newtonian blood flow by the Casson model [6–10]. In our work, we consider the K-L model because it is more effective than the Casson model. The yield stress (*τ*_*c*_) and the plasma viscosity (*k*_2_) are considered in the K-L model but only the yield stress (*τ*_*c*_) is concerned in the Casson model [11]. The artery is assumed to be long enough so the entrance and the end effects can be neglected. A cylindrical polar coordinate (*r*, *ψ*, *z*) is used to analyze the behavior of blood flow, where *r* and *z* are the radial and axial directions, respectively, and *ψ* is the azimuthal angle.

**Figure 1:**
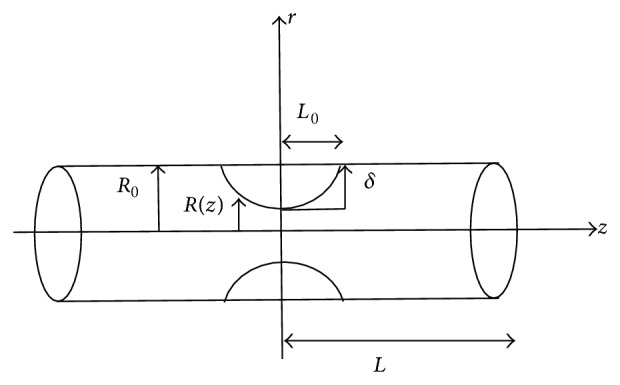
Geometry of arterial segment with stenosis.

**Figure 2:**
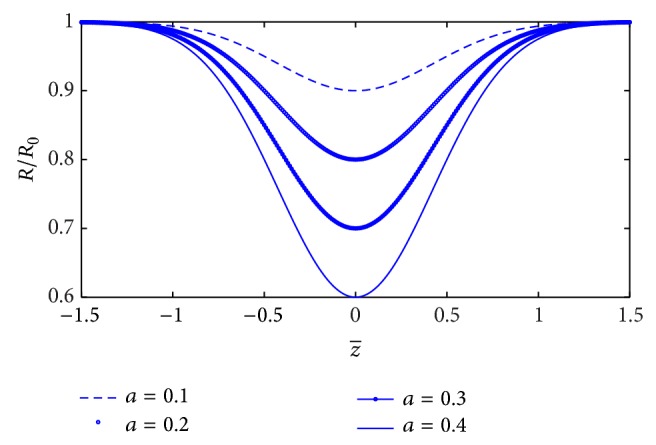
Stenosis geometries for different values of stenosis height *a*.

**Figure 3:**
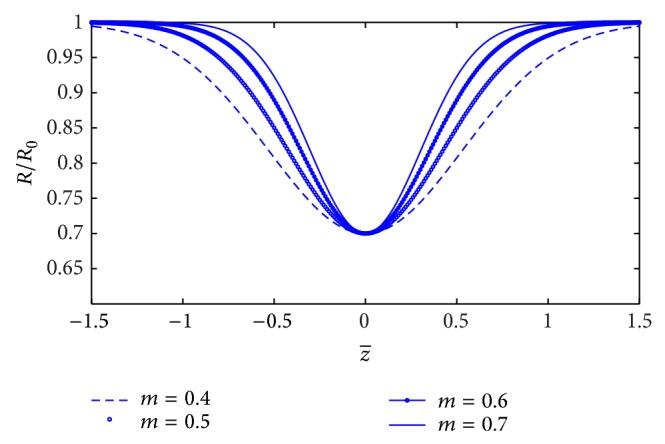
Stenosis geometries for different values of the stenosis shape parameter *m*.

Since the blood flow in narrow arteries is slow, the magnitude of the inertial forces is negligible, and the inertial terms in the momentum equations are neglected. The radial component of momentum equation is ignored because the considered flow is unidirectional. Therefore, the axial component of momentum equation is simplified to the following:

(1) $$\begin{array}{c} -\frac{dP}{dz}=\frac{1}{r}\frac{d\left(r\tau \right)}{dr}, \end{array}$$

where *P* is the pressure and *τ* is the shear stress. The K-L model that is a relationship between shear and strain rate is defined as follows:

(2) −dudr=f(τ)=2k22−4k1(τc−τ)−2k2k22−4k1(τc−τ)4k12,τ≥τc0,τ<τc,

where *u* is the velocity of blood in the axial direction, *τ*_*c*_ is the yield stress, *k*_1_ is plasma viscosity, and *k*_2_ is a parameter constant in K-L model. In this work, the geometry of segment of the narrow artery with mild bell-shaped stenosis is shown in Figure 1 and defined as follows:

(3) $$\begin{array}{c} R\left(z\right)=R_{0}\left(1-ae^{-bz^{2}}\right), \end{array}$$

where *R*(*z*) is the radius of artery in the stenosis region and *R*_0_ is radius of normal artery. Note that *a* is a nondimensional parameter of stenosis height, defined as *a* = *δ*/*R*_0_, where *δ* is depth of stenosis. Parameter *b* is a nondimensional parameter that is the length of the stenosis in the segment of narrow artery, defined as *b* = *m* ^2^/*L*_0_^2^, where *m* is the stenosis shape. When parameter *a* is variable and *b* is constant, the *R*/*R*_0_ marginally increase along the *z*-axis with decrease of *a* (Figure 2). On the other hand, keep *a* as constant and *b* as variable (for different values of *m* and fixed value of *L*_0_ = 1.5); it is noticed that the width of the stenosis (*R*/*R*_0_) increases with increase in value of *m* (Figure 3).

## 3. Model Analysis

Equation (1) can be solved with the assumption of no-slip boundary condition:

(4) $$\begin{array}{c} u=0\;\;\text{at}\;\;r=R\left(z\right), \end{array}$$

and the regularity condition:

(5) $$\begin{array}{c} \tau \;\;\text{is}\;\;\text{finite}\;\;\text{at}\;\;r=0. \end{array}$$

Integrating (1) and using (5), we obtain

(6) $$\begin{array}{c} \tau =-\frac{r}{2}\frac{dP}{dz}. \end{array}$$

The skin friction *τ*_*R*_ is obtained from (6) as

(7) $$\begin{array}{c} \tau_{R}=-\frac{R}{2}\frac{dP}{dz}. \end{array}$$

The volumetric flow rate is defined as follows:

(8) $$\begin{array}{c} Q=\frac{\pi R^{3}}{\tau_{R}}\overset{\tau_{R}}{\underset{0}{\int }}\tau^{2}f\left(\tau \right)d\tau , \end{array}$$

where *τ* and *τ*_*R*_ are defined in (6) and (7), respectively.

Substituting (2) into (8), we get

(9) Q=πR3τR×∫0τRτ22k22−4k1τc−τ−2k2k22−4k1τc−τ4k12dτ.

Integrating (9) and then simplifying, we obtain

(10) Q=πR34k122k23−4k1τc3−τR4+234k1τc−k222τR3+k223dd−k224k1227τR+454k1τc−k22τR+234k1τc−k222τR3+k223ddddddddd+234k1τc−k222τR3+k223.

Since $1/\sqrt{\tau_{R}}\ll 1$ and $1/\sqrt{{\tau_{R}}^{3}}\ll 1$, neglect the fourth and fifth terms of (10). The expression of flow rate is obtained as

(11) $$\begin{array}{c} Q=\frac{\pi R^{3}}{4{k_{1}}^{2}}\left\{\frac{2k_{2}}{3}-4k_{1}\left(\frac{\tau_{c}}{3}-\frac{\tau_{R}}{4}\right)-\frac{{k_{2}}^{2}}{4{k_{1}}^{2}}\left(\frac{2}{7}\sqrt{\tau_{R}}+\frac{{k_{2}}^{2}}{3}\right)\right\}. \end{array}$$

Solving (11) for *τ*_*R*_ and then simplifying, we get

(12) $$\begin{array}{c} \tau_{R}=\frac{5}{784}\left(\frac{{k_{2}}^{4}}{{k_{1}}^{6}}\right)-g\left(R\right)+\frac{{k_{2}}^{2}}{14{k_{1}}^{3}}\sqrt{{\left(\frac{k_{2}}{14k_{1}}\right)}^{2}-g\left(R\right)}, \end{array}$$

where *g*(*R*) = (2/*k*_1_)((2*k*_2_ − 4*k*_1_ *τ*_*c*_ + *k*_2_^2^)/3 − 4*Qk*_1_^2^/*πR* ^3^).

### 3.1. Skin Friction

Skin friction is friction of blood that is against the artery membrane. The expression of skin friction is obtained in (12). In the absence of any stenosis artery (*R*(*z*) = *R*_0_), the expression of skin friction becomes the following:

(13) $$\begin{array}{c} \tau_{N}=\frac{5}{784}\left(\frac{{k_{2}}^{4}}{{k_{1}}^{6}}\right)-g\left(R_{0}\right)+\frac{{k_{2}}^{2}}{14{k_{1}}^{3}}\sqrt{{\left(\frac{k_{2}}{14k_{1}}\right)}^{2}-g\left(R_{0}\right)}. \end{array}$$

The dimensionless form of skin friction with effect on stenosis artery is defined as the ratio between the skin friction of stenosis artery and the skin friction of normal artery. From (12) and (13), the dimensionless skin friction with effect of stenosis artery is obtained as

(14) τ1¯=τRτN=R0R3×5R3784k24k16−R3g(R)k214k12−g(R)+k22R0314k13k214k12−gR0−1dddsd+ k22R314k13k214k12−g(R)ddsd×5R03784k24k16−R03g(R0)k214k12−gR0dddsddd+k22R0314k13k214k12−gR0−1.

The dimensionless skin friction with the effect of stenosis artery and non-Newtonian behavior of blood is defined as the ratio between the skin friction of stenosis artery and the skin friction of Newtonian blood in normal artery. The expression of dimensionless skin friction on the effect of non-Newtonian blood is obtained as follows:

(15) τ2¯=τRτNe=R0R3×5R3784k24k16−R3g(R)k214k12−g(R)+k22R0314k13k214k12−g∗R−1dddsd+k22R314k13k214k12−g(R)ddd×5R03784k24k16−R03g∗(R)k214k12−g∗Rddddff+k22R0314k13k214k12−g∗R−1,

where the skin friction of Newtonian blood in stenosis artery is defined as

(16) $$\begin{array}{c} \tau_{\text{Ne}}=\frac{5}{784}\left(\frac{{k_{2}}^{4}}{{k_{1}}^{6}}\right)-g^{*}\left(R\right)+\frac{{k_{2}}^{2}}{14{k_{1}}^{3}}\sqrt{{\left(\frac{k_{2}}{14k_{1}}\right)}^{2}-g^{*}\left(R\right)}, \end{array}$$

where *g* ^*^(*R*) = (2/*k*_1_)((2*k*_2_ + *k*_2_^2^)/3 − 4*Qk*_1_^2^/*πR* ^3^).

### 3.2. Resistance of Blood Flow

The resistance of blood (*λ*) flow is the ratio of pressure difference across the artery circuit and the blood flow rate that is defined as

(17) $$\begin{array}{c} \lambda =\frac{P_{2}-P_{1}}{Q}, \end{array}$$

where *P*_1_ and *P*_2_ are pressure of input and output blood in artery circuit, respectively, and *Q* is blood flow rate that is given in (11).

Using (12) into (7), we get

(18) $$\begin{array}{c} -\frac{dP}{dz}=\frac{10}{784R}\left(\frac{{k_{2}}^{4}}{{k_{1}}^{6}}\right)-\frac{2g\left(R\right)}{R}+\frac{{k_{2}}^{2}}{7R{k_{1}}^{3}}\sqrt{{\left(\frac{k_{2}}{14k_{1}}\right)}^{2}-g\left(R\right)}. \end{array}$$

Integrating (18) along the length of the artery and using the condition that *P* = *P*_1_ at *z* = −*L* and *P* = *P*_2_ at *z* = *L*, we obtain

(19) P1−P2=10784Rk24k16∫−LLdz(R/R0)−2∫−LLg(R)Rdz+k227k13∫−LL1Rk214k12−gRdz.

Simplifying (19), we can obtain the following expression for pressure drop:

(20) P1−P2=20784Rk24k16×L−L0+∫0L0dz(R/R0)−4L−L0gR0R0+∫0L0gRRdz+2k227k13(L−L0)1R0k214k12−g(R0)fffffifff+∫0L01Rk214k12−gRdzk214k12−gR0.

The resistance of blood flow for K-L model in a stenosis artery is obtained as

(21) λ=20784QRk24k16L−L0+∫0L0dz(R/R0)−4Q(L−L0)g(R0)R0+∫0L0g(R)Rdz+2k227Qk13(L−L0)1R0k214k12−g(R0)ddddidddd+∫0L01Rk214k12−gRdz.

In the absence of constriction, the resistance of blood flow in normal artery is given as follows:

(22) λN=2LR0Q ×10784k24k16−2gR0+k227k13k214k12−gR0.

The nondimensional resistance flow of blood that is a ratio between the resistance of blood in stenosis and normal artery is obtained as

(23) λ1¯=λλN=1−L0L+1L×10784k24k16I1−2R0I2+R0k227k13I3k227k13k214k12−gR0−1ddd×10784k24k16−2gR0k214k12−gR0ssssssssss+k227k13k214k12−gR0−1,

where *I*_1_ = ∫_0_^*L*_0_^(*dz*/(*R*/*R*_0_)),  *I*_2_ = ∫_0_^*L*_0_^(*g*(*R*)/*R*)*dz*, and $I_{3}=\int_{0}^{L_{0}}\left(1/R\right)\sqrt{{\left(k_{2}/14k_{1}\right)}^{2}-g(R)}dz$.

Substitute *R* and *g*(*R*) into the integrals *I*_1_,  *I*_2_, and *I*_3_. The integrals are reduced to the following:

(24) I1=∫0L0dz(1−ae−bz2),I2=∫0L022k2−4k1τc+k223−4Qk12πR031−ae−bz23ddddd×2k2−4k1τc+k223−4Qk12πR031−ae−bz23k1R01−ae−bz2−1dz,I3=∫0L01R0(1−ae−bz2)sss×k214k12−2k1−4Qk12πR031−ae−bz231/2ssssssdss×2k2−4k1τc+k2234Qk12πR031−ae−bz23ssssssssdssssss−4Qk12πR031−ae−bz231/2dz.

In order to compute *I*_1_,  *I*_2_, and *I*_3_, we use a two-point Gauss quadrature formula. The integrals are evaluated as follows:

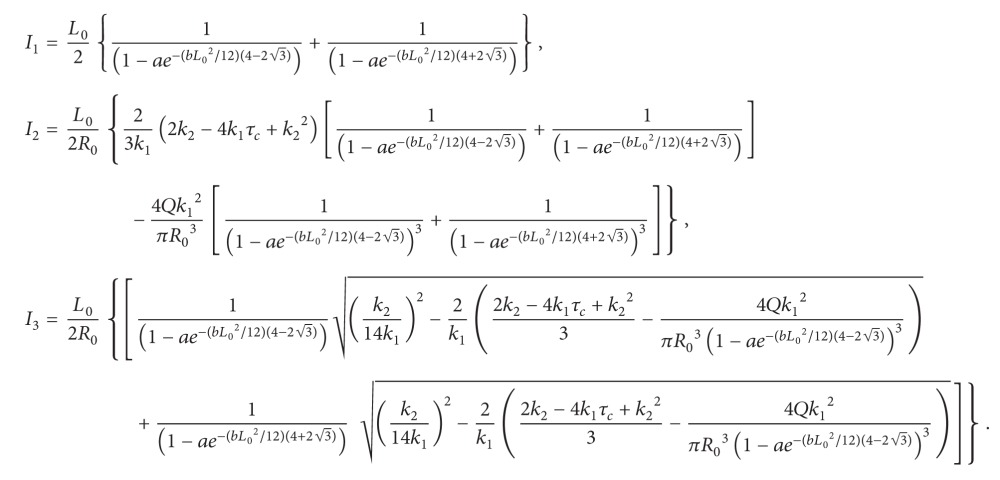
(25)
We normalize the resistance of blood flow for K-L model (*λ*) by the resistance of Newtonian blood in stenosis artery (*λ*_Ne_):

(26) λ2¯=107841−L0L+I1L−2Qg(R0)1−L0L+I2L1−L0L1R0k214k12−g(R0)+I2Lsssss+R0k227k131−L0L1R0k214k12−g(R0)ssssssssssssdssss×k214k12−g(R0)+I2L ×10784k24k16−2g∗R+k227k13k214k12−g∗R−1,

where the resistance of Newtonian blood in normal artery is given as

(27) λNe=2LR0Q ×10784k24k16−2g∗R0+k227k13k214k12−g∗R0.

## 4. Numerical Simulations of Results

The objective of our study is to discuss the effect of various parameters on the physiologically important flow quantities such as flow rate, skin friction, and resistance of blood flow. The ranges of parameters are shown in Table 1 that is according to Misra and Shit [15] and Venkatesan et al. [16].

### 4.1. Blood Flow Rate

**Figure 4:**
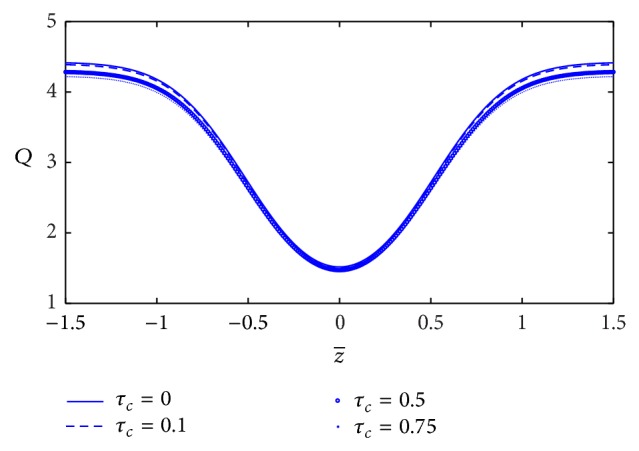
Variation of blood flow rate with axial distance for the different values of the yield stress.

**Figure 5:**
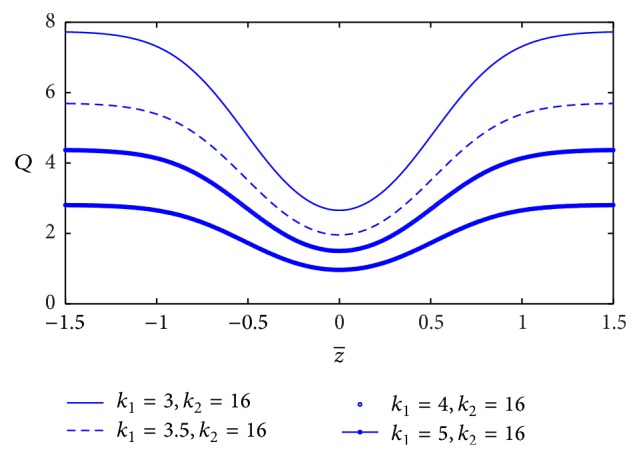
Variation of blood flow rate with axial distance for the different values of plasma viscosity.

Figure 4 shows the variation of blood flow rate (*Q*) with axial distance *z* for the different values of the yield stress (*τ*_*c*_). We can notice that the blood flow rate decreases very slightly with increase of the yield stress. The variation of blood flow rate with *z*-axis for different values of plasma viscosity coefficient (*k*_1_) is plotted in Figure 5. It is observed that the blood flow rate decreases marginally with increase of plasma viscosity coefficient.

The blood flow rate in narrow artery decreases very slightly with the increase of yield stress, but it decreases significantly with the increase of plasma viscosity coefficient. In the Casson and the Herschel-Bulkley model, the blood flow rate also decreases with the increase of yield stress [15, 16]. We conclude that the effect of yield stress in blood flow rate is consistent in three models (K-L, Casson, and Herschel-Bulkley). However, not only the yield stress but also the plasma viscosity affects the blood flow rate in the K-L model. The blood flow rate decreases significantly with the increase of plasma viscosity coefficient.

### 4.2. The Skin Friction

#### 4.2.1. The Effect of Stenosis on Skin Friction

**Figure 6:**
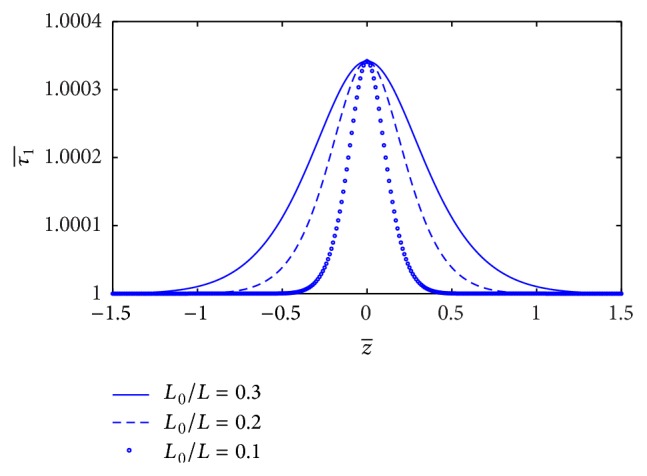
Variation of skin friction $\bar{\tau_{1}}$ with axial distance for the different values of *L*_0_/*L*.

**Figure 7:**
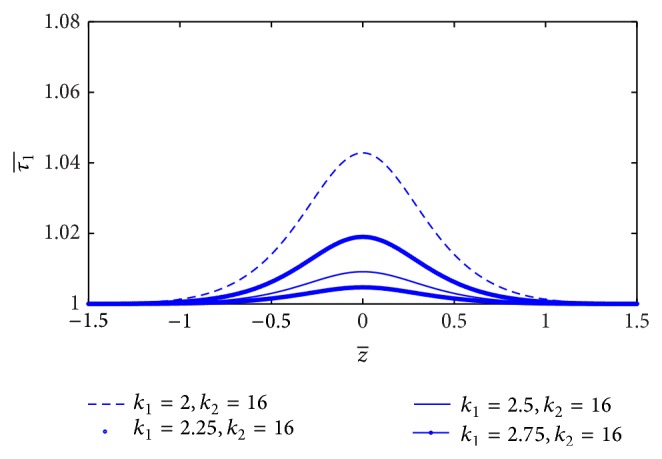
Variation of skin friction $\bar{\tau_{1}}$ with axial distance for the different values of plasma viscosity.

The estimation of skin friction $\left(\bar{\tau_{1}}\right)$ along the axial direction *z* for different values of *L*_0_/*L* is sketched in Figure 6. It is indicated that skin friction $\left(\bar{\tau_{1}}\right)$ increases significantly with the increase of stenosis length (*L*_0_). The variation of skin friction $\left(\bar{\tau_{1}}\right)$ with *z*-axis for different values of plasma viscosity coefficient (*k*_1_) is shown in Figure 7. It is noticed that the skin friction $\left(\bar{\tau_{1}}\right)$ decreases marginally with the increase of plasma viscosity coefficient.

**Figure 8:**
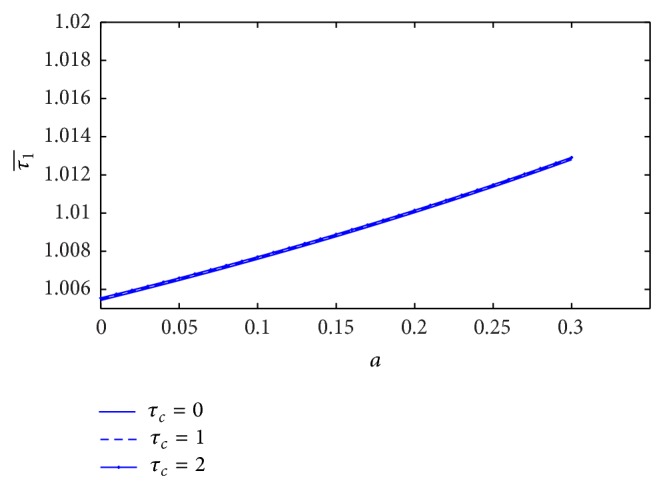
Variation of skin friction $\bar{\tau_{1}}$ with stenosis height *a* for the different values of yield stress.

**Figure 9:**
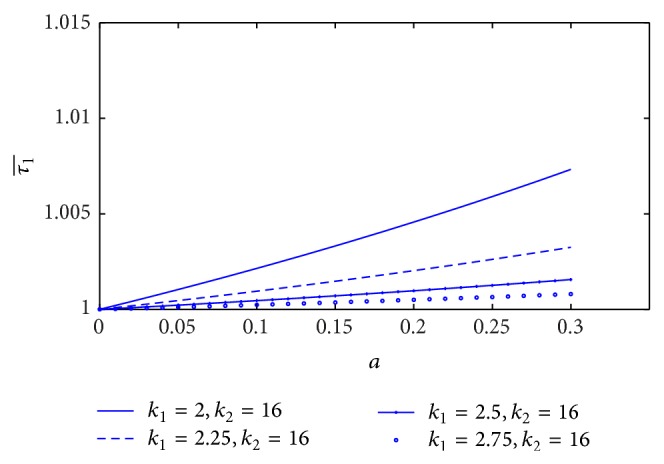
Variation of skin friction $\bar{\tau_{1}}$ with stenosis height *a* for the different values of plasma viscosity.

When we study the skin friction $\left(\bar{\tau_{1}}\right)$ at midpoint (*z* = 0), the variation of skin friction with stenosis height (*a*) for different values of the yield stress is sketched in Figure 8. It is observed that the skin friction increases very slightly with the increase of yield stress. On the other hand, the skin friction at the midpoint decreases significantly with increase of plasma viscosity coefficient that is shown in Figure 9.

The effect of stenosis on skin friction in the K-L model is that the skin friction $\left(\bar{\tau_{1}}\right)$ increases when either the stenosis length or yield stress increases, but it decreases with the increase of plasma viscosity coefficient. The skin friction increases significantly with the increase of stenosis length in the Casson model but it decreases marginally with the increase of yield stress in both of the Casson and the Herschel-Bulkley models [15, 16]. The effects of stenosis on skin friction in the K-L and the Cason model are in good agreement when we consider the variation of stenosis length.

#### 4.2.2. The Effect of Non-Newtonian Behavior on Skin Friction

**Figure 10:**
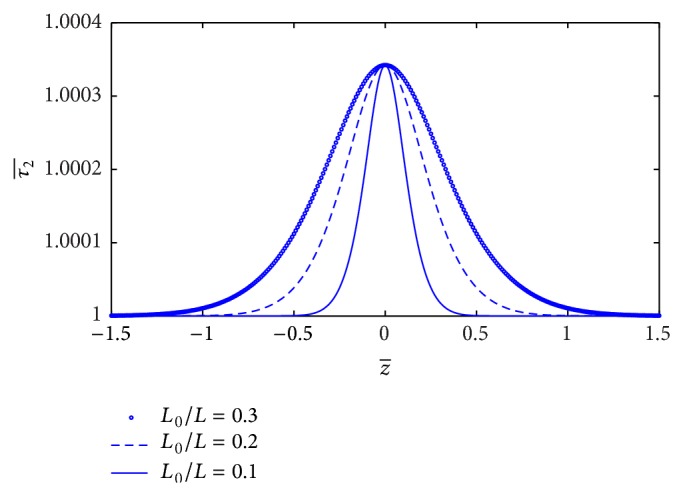
Variation of skin friction $\bar{\tau_{2}}$ with axial distance for the different values of *L*_0_/*L*.

**Figure 11:**
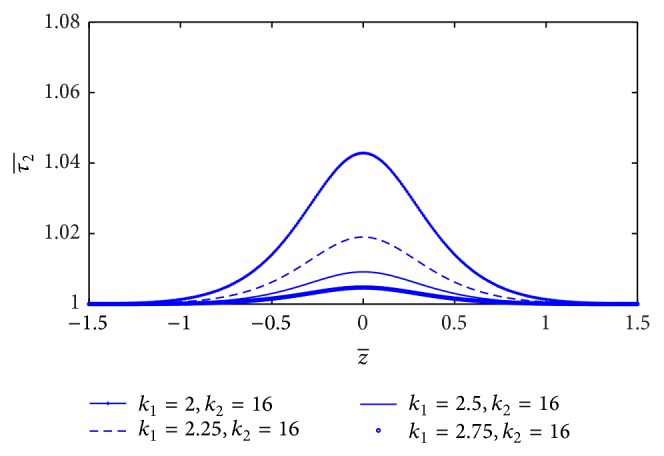
Variation of skin friction $\bar{\tau_{2}}$ with axial distance for the different values of plasma viscosity.

**Figure 12:**
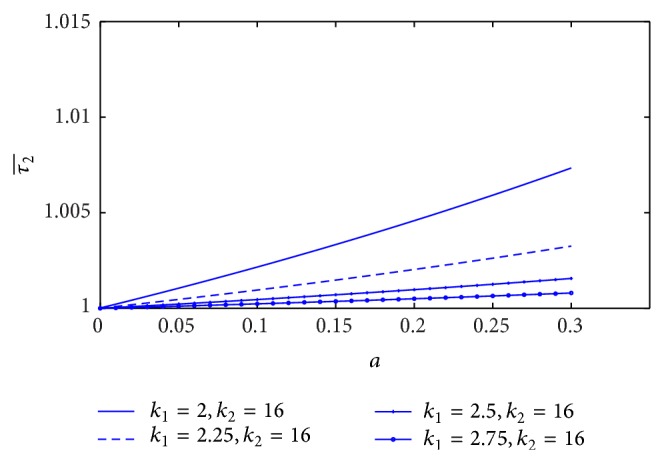
Variation of skin friction $\bar{\tau_{2}}$ with stenosis height *a* for the different values of plasma viscosity.

Many researchers studied the Newtonian blood flow. Since the blood behaves as non-Newtonian fluid at the low shear rate, we would like to know how the non-Newtonian blood behavior affects the skin friction of artery. The variation of skin friction $\left(\bar{\tau_{2}}\right)$ along the *z*-axis for different values of *L*_0_/*L* is plotted in Figure 10. It is indicated that skin friction $\left(\bar{\tau_{2}}\right)$ increases significantly when the stenosis length increases. By our simulation, the yield stress does not affect the skin friction in non-Newtonian behavior that is consistent with the Casson model [16].  Figure 11 shows the plot of skin friction $\left(\bar{\tau_{2}}\right)$ along the *z*-axis for the different values of plasma viscosity coefficient. It is noticed that skin friction $\left(\bar{\tau_{2}}\right)$ decreases marginally with increase of plasma viscosity coefficient. The plot skin friction at midpoint of stenosis with stenosis height (*a*) for the different values of plasma viscosity coefficient is shown in Figure 12. It informs that the skin friction decreases linearly with increase of plasma viscosity coefficient.

The effect of non-Newtonian behavior on skin friction in the K-L model is that the skin friction $\left(\bar{\tau_{2}}\right)$ increases significantly with the increase of stenosis length, but it decreases with increase of the plasma viscosity coefficient. For the Casson model, the skin friction increases significantly with the increase of stenosis length that is identical with the K-L model [16] but is not consistent with the Herschel-Bulkley model [15]. Not only the stenosis length but also the plasma viscosity affects the skin friction of non-Newtonian fluid in the K-L model.

### 4.3. The Resistance of Blood Flow

#### 4.3.1. The Effect of Stenosis on the Resistance of Blood Flow

**Figure 13:**
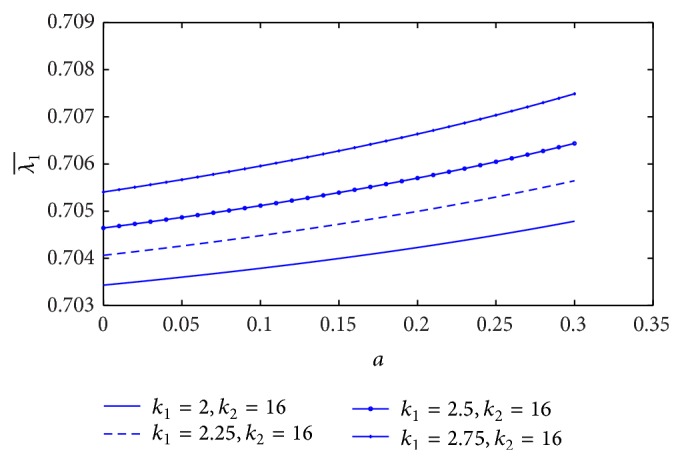
Variation of resistance of blood flow $\bar{\lambda_{1}}$ with stenosis height *a* for the different values of plasma viscosity.

**Figure 14:**
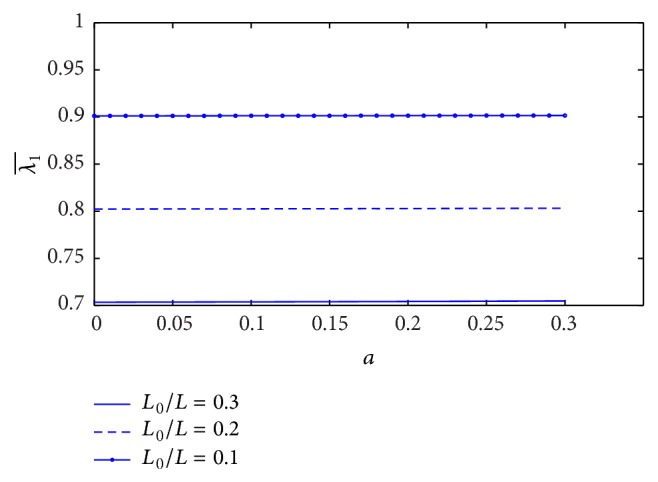
Variation of resistance of blood flow $\bar{\lambda_{1}}$ with stenosis height *a* for the different values of *L*_0_/*L*.

**Figure 15:**
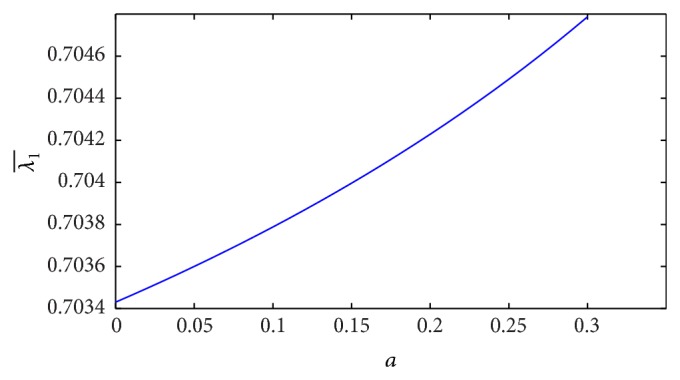
Variation of resistance of blood flow $\bar{\lambda_{1}}$ with stenosis height *a* for *L*_0_/*L* = 0.1.

**Figure 16:**
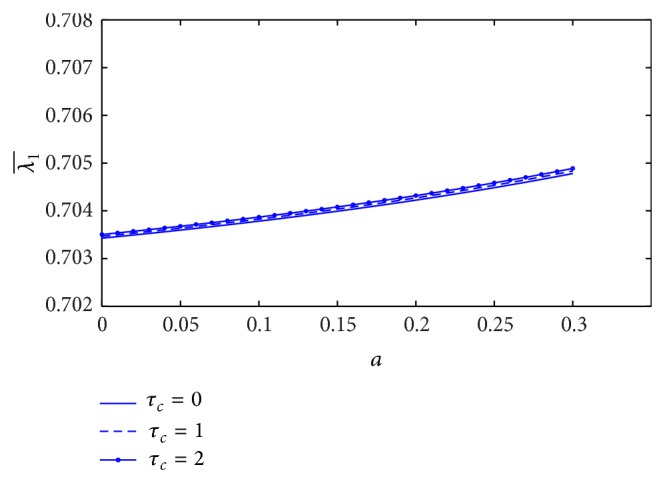
Variation of resistance of blood flow $\bar{\lambda_{1}}$ with stenosis height *a* for the different values of yield stress.

Figure 13 shows the variation of resistance of blood flow $\left(\bar{\lambda_{1}}\right)$ with the height of stenosis (*a*) for different values of plasma viscosity coefficient. It is noticed that the resistance of blood flow $\left(\bar{\lambda_{1}}\right)$ increases marginally with the increase of plasma viscosity coefficient. The variation of resistance of blood flow $\left(\bar{\lambda_{1}}\right)$ with the height of stenosis for different values of *L*_0_/*L* is sketched in Figure 14. It is observed that the resistance of blood flow $\left(\bar{\lambda_{1}}\right)$ decreases significantly with increase of stenosis length. We can notice that the resistance of blood flow $\left(\bar{\lambda_{1}}\right)$ seems constant along the *z*-axis for fixed *L*_0_/*L*, but the resistance of blood flow increases linearly along the *z*-axis when the plot is sketched in Figure 15. The estimation of resistance of blood flow $\left(\bar{\lambda_{1}}\right)$ with the height of stenosis for the different yield stress is shown in Figure 16. The resistance of blood flow $\left(\bar{\lambda_{1}}\right)$ increases slightly when the yield stress increases.

In the K-L model, the effect of stenosis on the resistance of blood flow is that the resistance of blood flow $\left(\bar{\lambda_{1}}\right)$ increases when either the plasma viscosity coefficient or yield stress increases, but it decreases with the increase of stenosis length. In the Casson model, resistance of blood flow increases with the increase of stenosis length or yield stress which is the same as the K-L model [16]. Moreover, the resistance of blood flow increases marginally with the increase of plasma viscosity coefficient in the K-L model.

#### 4.3.2. The Effect of Non-Newtonian Blood Behavior on the Resistance of Blood Flow

**Figure 17:**
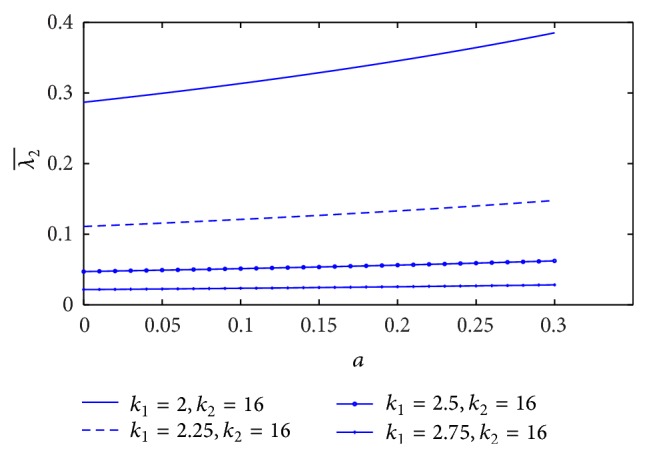
Variation of resistance of blood flow $\bar{\lambda_{2}}$ with stenosis height *a* for the different values of plasma viscosity.

**Figure 18:**
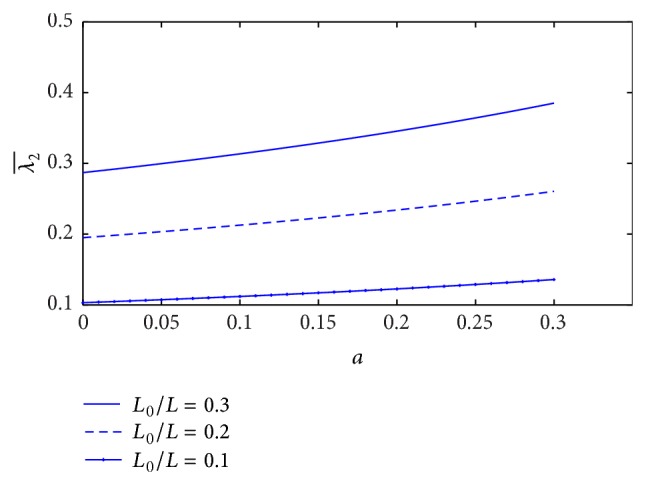
Variation of resistance of blood flow $\bar{\lambda_{2}}$ with stenosis height *a* for the different values of *L*_0_/*L*.

**Figure 19:**
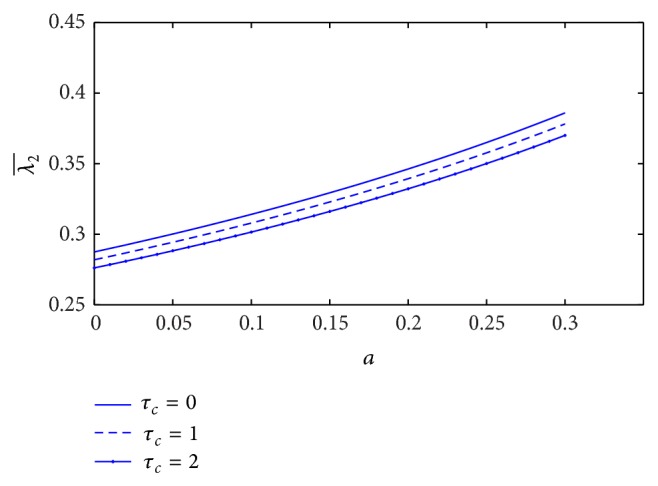
Variation of resistance of blood flow $\bar{\lambda_{2}}$ with stenosis height *a* for the different values of yield stress.

The plot of resistance of blood flow $\left(\bar{\lambda_{2}}\right)$ with the height of stenosis (*a*) for different values of plasma viscosity coefficient is sketched in Figure 17. It is indicated that the resistance of blood flow $\left(\bar{\lambda_{2}}\right)$ decreases marginally with increase of plasma viscosity coefficient.  Figure 18 shows the variation of resistance of blood flow $\left(\bar{\lambda_{2}}\right)$ with height of stenosis (*a*) for the different values of *L*_0_/*L*. It is informed that resistance of blood flow $\left(\bar{\lambda_{2}}\right)$ increases significantly with the increase of stenosis length. The variation of resistance of blood flow $\left(\bar{\lambda_{2}}\right)$ with height of stenosis (*a*) for different values of yield stress is plotted in Figure 19. We found that the resistance of blood flow $\left(\bar{\lambda_{2}}\right)$ decreases marginally with increase of yield stress.

The effect of non-Newtonian blood behavior on the resistance of blood flow in the K-L model is that the resistance of blood flow $\left(\bar{\lambda_{2}}\right)$ decreases when either the plasma viscosity coefficient or the yield stress increases, but it increases significantly with increase of the stenosis length. In the Casson model, the resistance of blood flow increases significantly with the increase of yield stress but it decreases with increase of stenosis length [16]. We found that the effect of non-Newtonian blood behavior on the resistance of blood flow in the K-L model is contradicted with the Casson model. However, the resistance of blood flow decreases with the increase of plasma viscosity (K-L model) or the increase of stenosis length (Casson model). Therefore, the plasma viscosity influences the resistance of blood flow in non-Newtonian fluid (K-L model).

## 5. Conclusion

Our study analyzed the steady flow of blood in a narrow artery with bell-shaped mild stenosis that treats blood as non-Newtonian fluid by using the K-L model. The numerical results are compared with the results of Venkatesan and coworkers, the Casson model [16], and also with results of Misra and Shit, the Herschel-Bulkley fluid model [15]. The main findings of our work are as follows.

- The blood flow rate in narrow artery in three models decreases very slightly with the increase of yield stress. But it decreases significantly with the increase of plasma viscosity coefficient in the K-L model.
- The effect of stenosis on skin friction is corresponding to the effect of non-Newtonian fluid on skin friction as follows.
  1. When the stenosis length (K-L and Casson model) increases, the skin friction increases significantly. On the other hand, the skin friction decreases slightly in Herschel-Bulkley and Casson model with the increase of yield stress.
  2. When the plasma viscosity coefficient increases in the K-L model, the skin friction decreases.
- The effect of stenosis on the resistance of blood flow in the K-L and the Casson model is consistent with the fact that the resistance of blood flow increases with the increase of yield stress but it decreases with the increase of stenosis length. When the plasma viscosity coefficient increases in the K-L model, the resistance of blood flow increases too.
- The effect of non-Newtonian blood behavior on the resistance of blood flow of the K-L model is contradicted with the Casson model. For the K-L model, the resistance of blood flow decrease when either the plasma viscosity coefficient or the yield stress increases, but it increase significantly with increase of the stenosis length.

The yield stress and the power law index are the most important parameters in the Casson and Herschel-Bulkley model, respectively. In the K-L model, the most important parameters are the plasma viscosity and yield stress. By our work, when we vary the plasma viscosity or yield stress, the important flow quantities (flow rate, skin friction, and resistance of blood flow) change significantly or slightly, respectively. We conclude that the K-L model is an improvement of Casson because it contains the most two important parameters (plasma viscosity and yield stress), whereas the Casson model takes into consideration only yield stress.

Our study provides the influence of the various parameters in flow quantities and compares with other models. We found that the stenosis length and the plasma viscosity influence the flow rate, the skin friction, and the resistance of blood flow. The flow quantities indicate where the stenosis is formed. Thus our work bears the potential to further explore the causes and development of arterial diseases such as atherosclerosis and cardiovascular.

## Figures and Tables

**Table 1:** The ranges of parameters.

Parameters	Values
Blood flow rate (*Q*)	0–5 cm^3^/s
Plasma viscosity (*k*_1_)	2.0–5.0 mpas
Constant parameter (*k*_2_)	16 (mpas)^1/2^
Stenosis shape (*m*)	0.2
Artery length (*L*)	5 cm
Radius of normal artery (*R*_0_)	1 mm
